# Online Quantitative Analysis of Perception Uncertainty Based on High-Definition Map

**DOI:** 10.3390/s23249876

**Published:** 2023-12-17

**Authors:** Mingliang Yang, Xinyu Jiao, Kun Jiang, Qian Cheng, Yanding Yang, Mengmeng Yang, Diange Yang

**Affiliations:** School of Vehicle and Mobility, Tsinghua University, Beijing 100084, China; sddxyml@126.com (M.Y.); jiaoxy13@163.com (X.J.); cq8625763@163.com (Q.C.); hustyyd@163.com (Y.Y.); ymm_kf@126.com (M.Y.)

**Keywords:** autonomous driving, perception uncertainty, high-definition map, uncertainty assessment

## Abstract

Environmental perception plays a fundamental role in decision-making and is crucial for ensuring the safety of autonomous driving. A pressing challenge is the online evaluation of perception uncertainty, a crucial step towards ensuring the safety and the industrialization of autonomous driving. High-definition maps offer precise information about static elements on the road, along with their topological relationships. As a result, the map can provide valuable prior information for assessing the uncertainty associated with static elements. In this paper, a method for evaluating perception uncertainty online, encompassing both static and dynamic elements, is introduced based on the high-definition map. The proposed method is as follows: Firstly, the uncertainty of static elements in perception, including the uncertainty of their existence and spatial information, was assessed based on the spatial and topological features of the static environmental elements; secondly, an online assessment model for the uncertainty of dynamic elements in perception was constructed. The online evaluation of the static element uncertainty was utilized to infer the dynamic element uncertainty, and then a model for recognizing the driving scenario and weather conditions was constructed to identify the triggering factors of uncertainty in real-time perception during autonomous driving operations, which can further optimize the online assessment model for perception uncertainty. The verification results on the nuScenes dataset show that our uncertainty assessment method based on a high-definition map effectively evaluates the real-time perception results’ performance.

## 1. Introduction

**Figure 1:**
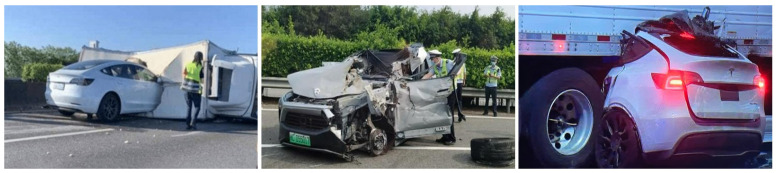
Autonomous driving accidents caused by perception uncertainty.

Autonomous driving (AD) holds immense significance in enhancing the safety and efficiency of road traffic [1]. Perception serves as a crucial means of obtaining environmental information, acting as a vital information source for the real-time operation of AD systems. The accuracy and completeness of perception results play a pivotal role in directly influencing the functional implementation of downstream modules, thereby impacting the safety of AD’s real-time operations. Figure 1 illustrates typical AD accidents caused by perception uncertainty in recent years, highlighting perception uncertainty as a significant factor contributing to accident risk. Perception uncertainty scenarios are commonplace due to internal and external factors that influence the perception system. These scenarios encompass various aspects, such as functional safety (FS) and the safety of the intended functionality (SOTIF). The presence of perception uncertainty directly affects the engineering practices of AD and represents a key issue that must be addressed in commercial operation [2,3]. Despite the considerable progress in enhancing perception performance in recent years, research on perception uncertainty and its associated risks has not garnered sufficient attention, and effective solutions remain a critical area in need of further exploration.

The real-time quantitative assessment of perception output uncertainty remains incomplete. Currently, scholars primarily assess uncertainty by offline comparisons between perception results and dataset annotations. This approach falls short in evaluating perception quality during real-time AD operations. Some researchers have introduced online evaluation methods for perception uncertainty, yet the complexity of AD introduces uncertainty through various factors, including sensors and perception algorithms. Existing studies focus on specific scenarios, functions, sensors, and algorithms, hindering the comprehensive evaluation of AD perception systems’ overall uncertainty. A unified analysis method and theory for addressing this issue are still in development.

High-definition maps (HD maps) store detailed information about roads, traffic signs, buildings, and environmental features. With a positional accuracy of up to 20 cm, a HD map also acts as a foundation for the fusion and cognition of AD perception [4]. HD maps boast high precision and a wealth of information, ensuring the reliable execution of AD functions while enhancing safety and efficiency. It is important to note that, currently, HD maps lack information on dynamic elements such as vehicles and pedestrians. Hence, assessing whether partial perception results can be matched and quality evaluated with the pre-existing map information during the real-time perception process of AD becomes pivotal. This approach aids in evaluating the uncertainty of the entire perception system’s output, significantly contributing to the improvement of accuracy and safety in AD.

There is a need for further research on how to effectively evaluate the real-time performance and uncertainty of perception results based on HD maps. This study introduces a novel real-time evaluation method for AD perception. The proposed uncertainty evaluation method, centered around a HD map, primarily focuses on unified deep neural network algorithms designed for multiple tasks. As deep neural network algorithms progress, they are evolving into unified models capable of handling various tasks. A single network can perform tasks such as object detection, semantic segmentation, and motion prediction. Despite the consistency in the internal architecture of the network, the output header is divided into multiple sections to accomplish distinct perception tasks. Given this unified approach, there exists a correlation between the uncertainty of perception in static elements and that in dynamic elements. Utilizing prior information from the HD map to assess the uncertainty of static elements in perception, and then mapping it to the uncertainty assessment of dynamic elements in perception provides a new approach to evaluating perception uncertainty.

Our contributions can be summarized as follows:

(1) The online uncertainty assessment model for static elements in perception was constructed based on a HD map. The model is capable of conducting real-time evaluations by integrating the lane line’s topological structure and pixel-level information. This integration allows for the assessment of both the existence uncertainty and spatial uncertainty associated with perception elements.
(2) The online uncertainty assessment model for dynamic elements in perception was constructed. The model leverages the online evaluation of static element uncertainty to infer dynamic element uncertainty. Based on a HD map, the online assessment of overall perception uncertainty was realized.
(3) A deep neural network model that performs online recognition of weather and scene factors, such as rain, snow, particulate matter, and illumination, was constructed. This model effectively identifies triggering factors for SOTIF and provides regulatory factors for the online assessment of uncertainty in perception elements, thereby enhancing the accuracy of the evaluation;
(4) We validated the perception uncertainty obtained using our method on the nuScenes dataset, demonstrating the correctness and accuracy of the proposed algorithm.

The rest of the paper is organized as follows: Section 2 provides an overview of the related works on perception uncertainties. In Section 3, we delve into the methodology employed for uncertainty evaluation. Section 4 presents the experimental results, and Section 5 concludes our findings and outlines potential avenues for future research.

## 2. Related Works

**Figure 2:**
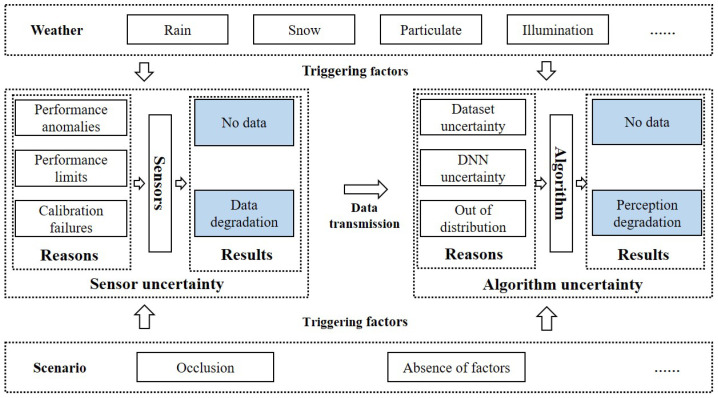
Autonomous driving perception and triggering factors of uncertainty.

In the data flow of the perception system, the perception sensors model the environment to acquire environmental information [5]. Subsequently, this undergoes data processing stages, including filtering and calibration, before the perception algorithm is applied. The algorithm then discerns environmental elements such as vehicles and pedestrians. The AD perception system comprises two fundamental components: sensors and perception algorithms, as shown in Figure 2. Influenced by external factors such as scenarios and weather, the perception system produces intricate results that encompass various uncertainties [6]. Quantitatively assessing the uncertainty of perception results forms the cornerstone for the effective utilization of these outcomes. Drawing on the above analysis, this paper encapsulates pertinent findings in the realm of uncertainty evaluation methods for perception sensors and perception algorithms.

### 2.1. Perception Sensor Uncertainty Evaluation Methods

Perception sensors, much like human eyes and ears, are capable of sensing environmental information [7]. Different sensors have distinct principles and advantages, making multi-source fused perception a crucial means of sensing [8]. From a functional safety perspective, sensors themselves may encounter performance obstacles and calibration failures. When anticipating SOTIF concerns, there may be performance deficiencies in sensors. Sensor faults can be manually inspected before the vehicle starts its journey and typically remain stable during real-time operation. However, performance deficiencies and calibration failures, triggered by external influences, can lead to momentary performance degradation. Thus, real-time monitoring is necessary. Scholars have conducted research on sensor failures from two perspectives: offline detection and online detection.

In offline detection methods for sensor performance, sensor failures are primarily assessed through performance tests conducted in laboratories or factories [9]. The assessment of sensor performance deficiencies involves conducting performance tests on a specific sensor under different weather conditions to determine the relationship between weather strength and sensor performance levels [10]. The detection of joint calibration failures in sensors relies on a collaborative calibration algorithm involving multiple sensors. It involves periodic manual inspection and the correction of sensor calibration relationships in the laboratory to achieve the detection of calibration relationship failures in sensors. This detection method is essentially a verification process for checking the effectiveness of joint sensor calibration.

In online detection, research methods include both model-based and data-driven methods [11,12]. For the parameters or features of the sensor’s raw data under study, the residual analysis method assesses whether the sensor is abnormal by comparing the evaluation results of sensor output with a set threshold [13]. The model updating method involves periodically updating and calibrating the perception model to detect abnormal sensor data. If the output result of a particular sensor data significantly differs from the output results of other sensor data, it can be considered that the sensor data are abnormal, requiring model updating [14]. The data-driven approach involves the real-time monitoring and analysis of changes in sensor data trends for anomaly detection. When a specific indicator of the sensor exceeds the predefined range, it can be determined that the sensor data are abnormal. In the early stages, sensor anomaly detection was primarily based on the inherent principles of sensors. This involved real-time monitoring of sensor data patterns, matching them with the threshold range of sensor performance data, and employing statistical analysis methods such as calculating the mean, variance, skewness, kurtosis, etc. to detect whether sensor data are abnormal [15]. Specifically, there is a wealth of research on algorithms for detecting failures in individual sensors. For instance, camera failure detection algorithms include image time series frames [16] and image quality assessments [17]. Evaluation methods for failures in lidar include spatio-temporal filtering [14] and random error models [18]. With the advancement of computer hardware technology, data-driven detection methods based on machine learning and statistical models have gained attention, such as support vector machines (SVMs) [19] and K-nearest neighbors (KNNs) [20]. These methods model and analyze sensor data to detect whether the sensor data are anomalous. Subsequently, deep learning algorithms have seen significant development, and many researchers utilize anomalous sensor data to train deep learning networks. These networks are then used for the online detection of sensor anomalies, including deep neural networks (DNNs), recurrent neural networks (RNNs), convolutional neural networks (CNN) [21].

In summary, offline detection methods for sensor uncertainty cannot meet the real-time operational requirements of AD. Online detection methods, on the one hand, excessively rely on the performance metrics of a specific category of sensors, lacking algorithmic transferability. On the other hand, evaluation methods based on deep learning depend on training datasets and struggle to address situations outside the distribution.

### 2.2. Perception Algorithm Uncertainty Evaluation Methods

Most literature studies on perception uncertainty are based on deep neural network perception algorithms. The DNN algorithm belongs to a “black box” model and is inexplicable [22]. The evaluation methods for perception uncertainty in AD mainly include offline evaluation and online evaluation [23,24].

When quantitatively evaluating perception uncertainty, the offline method primarily entails comparing the output results of neural networks with the dataset annotations to calculate errors. Nevertheless, implementing this approach on the open road during actual operation is impractical. The absence of annotations in the road dataset hinders the utilization of prior information for perception output results. Gawlikowski et al. [24] categorized uncertainty evaluation methods for deep neural networks into four types based on architectural essence and network quantity. These include single deterministic methods, Bayesian deep neural networks, ensemble methods, and detection data augmentation methods. The single deterministic method is comparatively efficient for evaluation, yet it heavily depends on the internal network architecture, dataset, and training process [25]. In terms of specific research methods, the Bayesian model proves adept at effectively capturing uncertainty, albeit at a higher computational cost [6,26,27]. Empirical evidence supports the efficacy of sampling-based uncertainty assessment methods, exemplified by Monte Carlo dropout (MC dropout) and deep ensemble (DE). In MC Dropout networks, probability is leveraged to deactivate neurons and refine the model. The output results can be approximated as randomly generated samples from the obtained posterior distribution [28]. Following a specific number of Monte Carlo samplings, the mean and variance of these samples can be utilized to approximate the probability distribution associated with cognitive uncertainty [29]. Compared to MC dropout, deep ensemble aimed at estimating cognitive uncertainty, all members follow the same architecture, with different parameter initializations, and are trained using the same overall but randomly shuffled training data, thus possessing the property of homogeneous heterogeneity [30]. During inference, similar to MC dropout, the output of each network in the deep ensemble is considered as independent samples of a mixed model. The mean and variance of these samples can then be used to approximate the probability distribution related to cognitive uncertainty. Through practical testing, it has been demonstrated that an ensemble of five networks is sufficient to approximate predictive probability distributions, enabling the online quantification assessment of uncertainty [31].

Therefore, it can be inferred that the cost and complexity of training a Bayesian model and MC dropout are both high, making it challenging to fulfill real-time evaluation requirements. For a specific deep neural network algorithm, the DE method can achieve an online uncertainty assessment of dynamic object detection (vehicles, pedestrians, etc.). However, the output results excessively rely on the distribution of results from the network itself, raising questions about the effectiveness of uncertainty assessment. This calls for objective clues other than the network itself to enhance the performance.

In summary, existing methods still cannot achieve the online evaluation of perception uncertainties, especially for dynamic objects. In fact, the perception sensors and algorithms of an AD system are intricately coupled. Isolating the study of one module alone does not allow for an effective evaluation of the comprehensive performance of the perception system. Consequently, conducting an uncertainty online assessment focusing on the perception output objects holds more profound academic significance in enhancing driving safety performance. Moreover, the HD map can provide prior information on static objects, which is an objective clue for inferring the uncertainty of detected dynamic objects. Thus, introducing prior perception information in the assessment of perception uncertainty can drive the real-time evaluation of perception, thereby facilitating the resolution of the aforementioned issues.

## 3. Methodology

**Figure 3:**
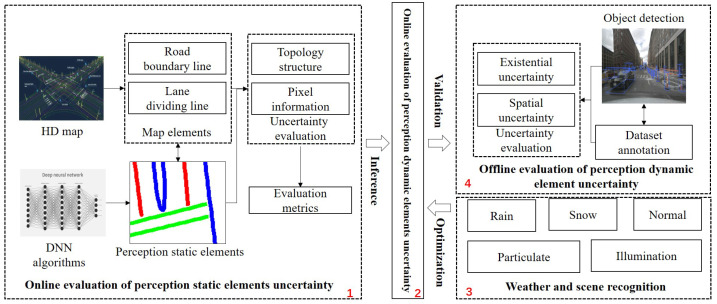
Quantitative evaluation architecture of perception uncertainty based on HD map. The red numbers 1–4 represent the four sections that this paper will cover, while the colored lines indicate perceptual static elements, such as lane lines.

The core of the online assessment of overall perception uncertainty is to use the uncertainty of static elements in perception to infer the uncertainty of dynamic perception elements. The online assessment of uncertainty in static elements in perception based on HD maps is fundamental, and its accuracy and completeness directly impact the effectiveness of the overall uncertainty assessment. By considering scene and weather factors, we can optimize the assessment method and greatly improve its accuracy in dealing with dynamic and static uncertainty. To further validate the effectiveness of the assessment method, it is necessary to obtain uncertainty information about dynamic perception elements. Therefore, a model for the offline uncertainty assessment based on dataset annotations was constructed. The logical architecture of the entire method is illustrated in Figure 3.

The online evaluation of uncertainty in static elements in perception utilizes the HD map as prior information, taking into account both the topological structure of environmental features and the pixel-level information of perception results. Scene and weather detection can identify the triggering factors of perception uncertainty. The offline assessment of perception uncertainty for dynamic elements based on dataset annotations mainly includes existential uncertainty and spatial uncertainty.

To more accurately convey the research approach of this paper, the following assumptions are made:

(1) This paper quantitatively assesses perception uncertainty based on the HD map, considering the HD map as accurate prior information. Therefore, this paper does not take into account the impact of the update frequency of the HD map;
(2) The perception DNN algorithm used in this paper is a multi-task unified network, meaning that the recognition of dynamic and static elements is performed by the same algorithm. Otherwise, the study on the correlation of uncertainty between dynamic and static elements in perception would be meaningless.

### 3.1. Selection of Environmental Feature Elements

**Figure 4:**
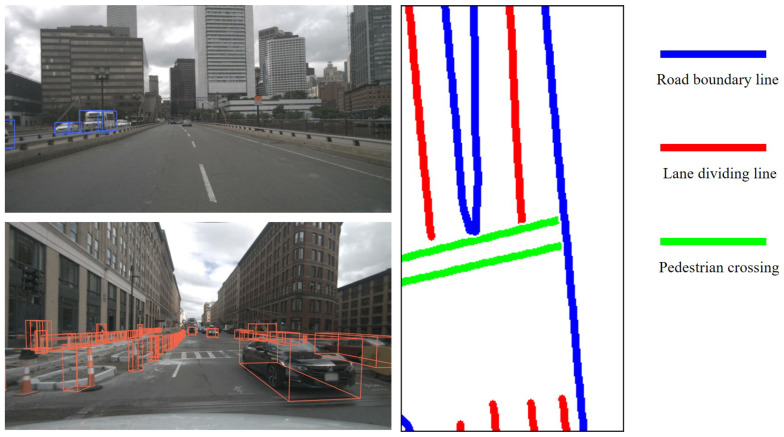
Environmental feature elements of the nuScenes dataset.

Environmental elements that do not frequently change over time are referred to as static environmental elements, such as lane lines and lampposts. On the other hand, if environmental elements undergo frequent changes over time, they are termed dynamic environmental elements, such as vehicles and pedestrians. In the context of AD perception, there are corresponding static elements in perception and dynamic elements in perception. The HD map encompasses an abundance of static environmental feature elements, comprising road boundary lines, lane dividing lines, and pedestrian crossings. Equation (1) summarizes the selection and representation of environmental feature elements.

(1) $$E=\left\{E_{s},E_{d}\right\}=\left\{\left\{E_{RL},E_{LL}\right\},\left\{E_{E},E_{S}\right\}\right\}$$

where $E_{s}$ denotes static environmental elements, while $E_{d}$ represents dynamic environmental elements. $E_{RL}$ and $E_{LL}$ denote road boundary lines and lane dividing lines within static feature elements. Moreover, $E_{E}$ and $E_{S}$ represent the existential and spatial information of perception results. The nuScenes dataset includes HD map data and annotated perception results, encompassing both static and dynamic elements of the environment [32]. The annotations of dynamic elements and the HD map serve as prior information for evaluating the perception output of AD and quantifying the uncertainty of the output results. This paper uses the nuScenes dataset as an example to showcase the presentation of selected environmental feature elements. Figure 4 illustrates the representation of dynamic and static elements in environmental perception.

In this paper, selected static elements comprise road boundary lines and lane dividing lines. The map encompasses various layers and offers robust methods for recording, rendering, and searching data on specific layers. Road boundary lines, for instance, serve to demarcate distinct road areas, with associated index information linked to the same segmentation line. Lane dividing lines demarcate distinct lanes within the same direction in a road area. The field of lane dividing lines comprises various nodes, each with its segmentation type, reflecting their physical characteristics. Given their enduring nature, these lines are qualified as static features. By amalgamating the characteristics of various static environmental factors, Equation (2) encapsulates the representation of static features.

(2) $$E_{s}=\left\{id,m,n,c_{pel}\right\}$$

where id denotes the index information of static map elements. Moreover, *m* and *n* represent the length and width dimensions of the static feature image, respectively. Additionally, the number of pixels $c_{pel}$ signifies the semantic information of each pixel lattice in the image.

The selected dynamic elements in this paper primarily encompass the outcomes of detected objects, including their semantic type, spatial position, bounding box size, orientation, and other relevant attributes. Equation (3) showcases the information attributes of dynamic elements in perception.

(3) $$E_{d}=\left\{id,c,\left(x,y,z\right),\left(w,l,h\right),r\right\}$$

where id denotes the index information of detected objects, *c* signifies the classification information for detected objects, and (x,y,z) represents the location information of detected objects. Additionally, (w,l,h) represents the size information of the bounding box for detected objects, and *r* stands for the orientation information of detected objects.

### 3.2. Online Assessment of Uncertainty in Static Elements in Perception

Initially, the static perception results in the DNN are aligned with the static elements in the HD map. The perception output of lane lines usually follows a linear distribution, represented by pixels in the image. A pixel-level uncertainty assessment is an effective approach for post-processing lane line perception because the lane line segmentation width in the nuScenes dataset is magnified by 10 times in the image. However, the method cannot account for the topological structure of lane lines. In cases of detection inaccuracy such as offset or rotation, pixel-level matching cannot well represent the lane detection performance. Therefore, this paper adopts a fusion approach of topological structure matching and pixel-level matching in the analysis of lane line uncertainties. This approach addresses both lane line omissions and the decrease in pixel matching accuracy under detection uncertainty.

#### 3.2.1. Uncertainty Evaluation Based on Pixel

**Figure 5:**
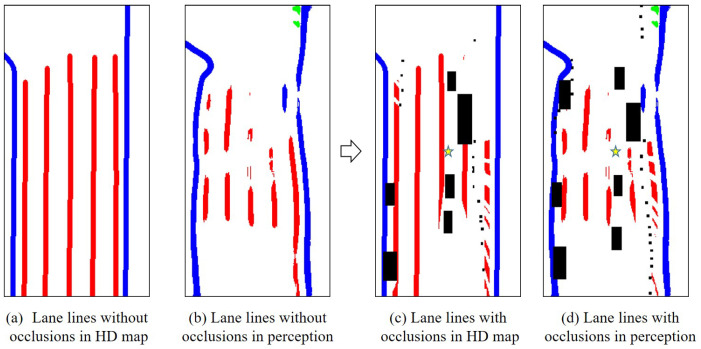
Before and after effects processing of occlusion handling (Frame 11). The arrow indicates the changes before and after the removal of occlusions, and the star represents the position of the ego vehicle after the occlusions are removed, providing a better description of the occlusion removal effect. The black boxes represent various dynamic objects.

The lane line topological structure ignores the impact of factors such as occlusion and absence. Therefore, the existential uncertainty of lane lines requires pixel matching of lane lines in the grid map. The lane lines in the HD map are complete and continuous, but in actual matching, it is necessary to remove the occluded parts. Therefore, this paper aims to address occlusion in the lane lines of the HD map to improve the accuracy of the IOU matching. As shown in Figure 5, the visual representation depicts the effects before and after occlusion handling in the process of image processing.

**Figure 6:**
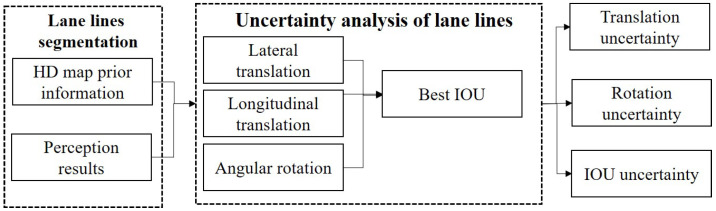
Uncertainty assessment based on lane line pixel-level.

Considering the slight global offset and rotation in the detection of lane lines and prior information, which results in a low accuracy of lane line matching, this paper adopts a local approach to consider lateral translation, longitudinal translation, and local rotation to find the best matching IOU, representing the detection effect in a local range. The translation and rotation values under the maximum IOU can serve as indicators of uncertainty in lane line detection. The relevant detection process is illustrated in Figure 6, and the specific implementation is illustrated in Algorithm 1.
**Algorithm 1** Lane line uncertainty assessment based on pixel-level.**Input:** The *i*th frame perception static result $P_{i}^{s}$, and the static feature result in the HD map       ${GT}_{i}^{s}$. **Output: **The optimal ${IOU}_{i}^{max}$ for static feature matching in the local range of the *i*-th frame.
  1:Assign an initial value to ${IOU}_{\max}$, ${IOU}_{max}$ = 0.  2:**for** $dx=-x_{set}$ to $x_{set}$ **do**  3:   **for** $dy=-y_{set}$ to $y_{set}$ **do**  4:     **for** $d\theta =-\theta_{set}$ to $\theta_{set}$ **do**  5:        Calculate the IOU for the current frame.  6:        **if** $IOU\geq {IOU}_{max}$ **then**  7:          Assign the IOU value to ${IOU}_{max}$ and output the corresponding dx, dy, dθ, $\sigma_{FN}$, $\sigma_{FP}$, at this moment.  8:        **else**  9:          ${IOU}_{max}$ remains unchanged.  10:        **end if**  11:     **end for**  12:   **end for**  13:**end for**  14:**return** The current frame’s ${IOU}_{i}^{max}$, along with the corresponding $dx_{i}$, $dy_{i}$, $d\theta_{i}$, $\sigma_{i}^{FN}$, $\sigma_{i}^{FP}$.


In a specific perception scene, the pixel matrix of perception results and HD map elements can be represented as m×n×3; where *m* signifies the number of row pixels, *n* denotes the number of column pixels, and 3 corresponds to the RGB channel. For each pixel, the respective values from the HD map and the corresponding values from the perception result are individually compared and recorded.

(4) $$\sigma_{FN}^{s}=\frac{N_{GT}-N_{TP}}{N_{GT}}$$

(5) $$\sigma_{FP}^{s}=\frac{N_{P}-N_{TP}}{N_{P}}$$

where $\sigma_{FN}^{s}$ and $\sigma_{FP}^{s}$ correspond to the miss rate and false rate, respectively, for static elements in perception at the pixel level. $N_{TP}$ represents the number of pixels in the current frame’s perception results where the static elements in perception successfully match those in the HD map. $N_{GT}$ denotes the number of pixels in the current frame’s perception results corresponding to static elements in the HD map. $N_{P}$ indicates the number of pixels in the current frame’s perception results for static elements in real-time perception results.

According to the calculation method of Algorithm 1 and Equation (5), the $IOU_{i}^{max}$ of the matching between local static elements in perception and the HD map can be obtained, along with the corresponding values for the miss rate $\sigma_{i}^{FN}$, false rate $\sigma_{i}^{FP}$, lateral offset $dx_{i}$, vertical offset $dy_{i}$, and angle rotation $d\theta_{i}$. This representation is used to denote the existence uncertainty and spatial uncertainty of static elements in perception.

(6) $$U_{s}^{IOU}=\left\{IOU_{i}^{max},\sigma_{i}^{FN},\sigma_{i}^{FP},dx_{i},dy_{i},d\theta_{i}\right\}$$

#### 3.2.2. Uncertainty Evaluation Based on Lane Line Topological Structure

**Figure 7:**
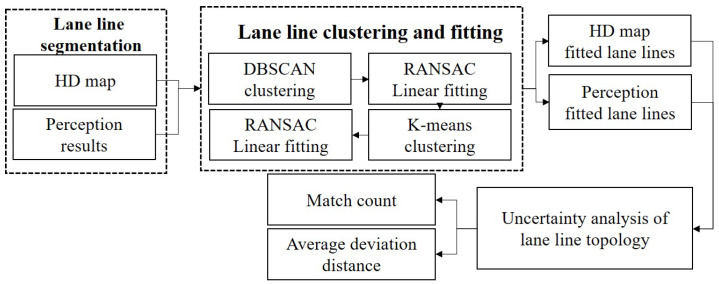
Uncertainty assessment based on lane line topological structure.

The flowchart for the uncertainty assessment based on the lane line topological structure is shown in Figure 7. This study was based on the nuScenes dataset, so post-processing was applied to the map lane line prior information and the perception lane lines from semantic segmentation in the images. Since the lane lines exhibited a linear distribution, the initial clustering was performed using the DBSCAN (density-based spatial clustering of applications with noise) clustering algorithm [33,34].

DBSCAN, which stands for density-based spatial clustering of applications with noise, is an algorithm that identifies clusters in spatial databases with noise by partitioning regions with sufficient density. It is capable of detecting clusters of arbitrary shapes and defines a cluster as the maximum set of density-connected points. For lane line clustering, this algorithm can perform longitudinal clustering along the distribution of lane lines, ensuring a fundamental clustering effect for lane lines. DBSCAN relies on a set of neighborhoods to describe the density of a sample set, with parameters (eps, min_samples) to characterize the density of the sample distribution within the neighborhood. In this context, ‘eps’ denotes the neighborhood distance threshold for a sample, and ‘min_samples’ indicates the threshold for the minimum number of samples in the neighborhood within a distance of ‘eps’ from a given sample. However, a single clustering algorithm may not achieve optimal results, and further optimization of the clustering results is needed. The algorithm for secondary clustering can be expressed as in the following.

Due to the lane lines’ pixel distribution being stripe-like, the fitting of lane lines can be achieved using the RANSAC (random sample consensus) linear fitting method [35]. RANSAC can iteratively estimate the parameters of a mathematical model from a set of observation data that may contain “outliers” in a dataset. Lane line perception can be intermittent due to factors like occlusion and lane line absence. Therefore, relying solely on DBSCAN may not produce satisfactory results in a single attempt. It is necessary to perform a secondary clustering on the previous clustering results. This paper further utilizes the K-means [36] clustering method for this secondary clustering, the specific implementation of clustering is illustrated in Algorithm 2.

**Figure 8:**
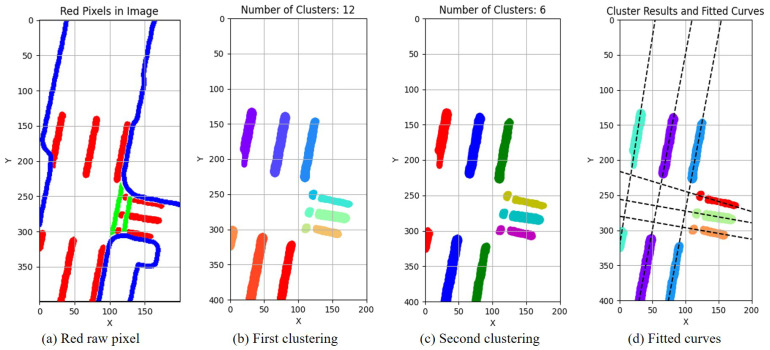
Lane lines clustering and fitting results of HD map (Frame 553). (**a**) The red pixels represent lane lines, and they need to be clustered and fitted, while pixels in blue and green are not considered. (**b**) After the first clustering, the lane lines form 12 clusters, represented by different colors. (**c**) After the second clustering, the lane lines form 6 clusters, represented by different colors. (**d**) After the second clustering, curves fitted to the lane lines.

**Figure 9:**
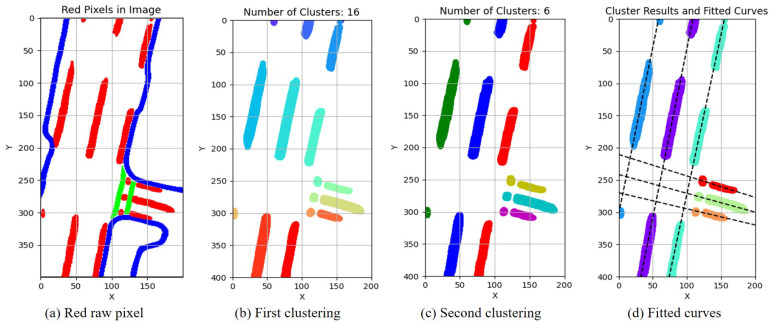
Lane lines clustering and fitting results of perception results (Frame 553).(**a**) The red pixels represent lane lines, and they need to be clustered and fitted, while pixels in blue and green are not considered. (**b**) After the first clustering, the lane lines form 16 clusters, represented by different colors. (**c**) After the second clustering, the lane lines form 6 clusters, represented by different colors. (**d**) After the second clustering, curves fitted to the lane lines.

Figure 8 and Figure 9 illustrate the process of clustering and fitting, and Figure 8a represents the detection results of lane lines, which are fragmented into scattered or banded points due to occlusion factors. The extraction and DBSCAN clustering of red pixels were performed, resulting in clustered outcomes as shown in Figure 8b, indicating the successful clustering of continuous lane lines. The further utilization of the K-means clustering method produced the results shown in Figure 8c, revealing the successful linear clustering of lane lines. Based on the final clustering outcome, the lane fitting results are depicted in Figure 8d. By comparing the clustering and fitting processes in Figure 8 and Figure 9, it can be observed that the proposed method in this paper effectively fits real static elements in perception such as lane lines. This lays the foundation for the uncertainty analysis of the HD map and perception results.
**Algorithm 2** Secondary clustering based on the K-means clustering method.**Input:** The clustering results $C_{1:N}$.
**Output:** Results after the secondary clustering $C_{1:M}$.
  1:**while** The number of clusters in $C_{1:N}$ > 0 **do**  2:   Find the individual cluster $C_{max}$ with the highest number of points in the clustering results.  3:   Remove the individual cluster $C_{max}$ from $C_{1:N}$.  4:   Perform RANSAC linear fitting on this largest cluster to obtain the slope (*m*) and intercept (*b*) of the line.  5:   **while** $C_{max}$ == $C_{max}^{new}$ **do**  6:     **for** *C* in $C_{1:N}$ **do**  7:        **for** *P* in cluster **do**  8:          Calculate the distance of every point *P* to the fitting curve  9:          Calculate the average distance from all points in a clustering result to the fitted curve.  10:        **end for**  11:     **end for**  12:     **if** average distance < threshold. **then**  13:        Merge this cluster with the largest cluster to generate the new $C_{max}^{new}$.  14:        Remove the cluster from $C_{1:N}$.  15:     **end if**  16:   **end while**  17:   Add $C_{max}^{new}$ to $C_{1:M}$  18:**end while**  19:**return**$C_{1:M}$.


For the fitted curves in the HD map and the perception results, the uncertainty assessment was performed. The specific algorithm is as follows: If two lines does not intersect, the distance between the lines is directly calculated. Otherwise, the average distance from 10 points on each side of the intersection point to the other line is calculated. If the average distance is less than a set threshold, the curve is considered to be the same lane line. A smaller average distance indicates lower uncertainty of the lane lines.

Based on the above analysis, uncertainty based on lane line topological structure in *i* th frame can be expressed in Equation (7):

(7) $$U_{s}^{line}=\left\{N_{i}^{M},N_{i}^{gt},N_{i}^{pred},{\Delta d}_{i}\right\}$$

where $N_{i}^{M}$ represents the number of successful lane line perception matches in the current frame, $N_{i}^{gt}$ represents the number of lane lines in the HD map, $N_{i}^{pred}$ represents the number of lane lines in the perception results, and ${\Delta d}_{i}$ represents the average offset error of selected points after successful lane line matching.

### 3.3. Online Assessment of Uncertainty in Dynamic Elements in Perception

Dynamic elements in perception object detection encompasses various entities such as cars, pedestrians, and temporary traffic facilities. As these environmental feature elements are absent in HD maps, HD maps cannot directly determine the uncertainty of dynamic elements in perception. Therefore, this paper constructs an online evaluation method that infers the uncertainty of dynamic elements based on the uncertainty of static elements, ultimately achieving an assessment of the overall perception uncertainty. To further validate the effectiveness of the method, it was necessary to combine an offline evaluation of the uncertainty of dynamic elements in perception with dataset annotations, serving as prior data to validate the method’s effectiveness. Therefore, this section mainly includes two parts: the construction of an online assessment model for the uncertainty of dynamic elements in perception, and an offline evaluation model for the uncertainty of dynamic elements in perception.

#### 3.3.1. Online Uncertainty Assessment Model Construction

This section primarily focuses on establishing the associative relationship between uncertainties in dynamic and static elements. It aims to leverage static prior information from the HP map to map and assess the overall perception uncertainty.

The correlation relationship of perception dynamic and static elements primarily analyzes existential uncertainty and spatial information uncertainty. Existential uncertainty encompasses both missed and false detections, while spatial uncertainty includes position, size, and orientation. In the analysis of static uncertainty perception, a comprehensive consideration is needed for the fusion of the topological structure and pixel-level uncertainty. Regarding the perception of dynamic elements, spatial uncertainty involves evaluating the degree of impact of the position, size, and orientation on driving risks. Among these factors, this paper posits that positional uncertainty exerts the most significant impact on perception uncertainty, followed by the size uncertainty, and finally the orientation uncertainty of perception objects. Through weighting, the total perception uncertainty can be computed in Equation (8).

(8) $$U_{P}=\sum_{i}\zeta_{i}\times E_{i},\;E\in U_{P}$$

where $\zeta_{i}$ denotes the weights assigned to different types of uncertainty in $U_{P}$. Next, we establish the relationship between the uncertainty of static elements in perception and dynamic elements in perception.

(9) $$\alpha =f(U_{d},U_{s})$$

where α represents the correlation of dynamic and static elements in perception.

Specifically, uncertainty relationships of the *i* th frame are established for each perception category as follows:

(10) $$\left\{U_{FN}:U_{FN}^{s}\to U_{FN}^{d}\right\}=\left\{\sigma_{FN}^{s}\times \left(N_{i}^{gt}/N_{i}^{M}\right)\to \sigma_{FN}^{d}\right\}$$

(11) $$\left\{U_{FP}:U_{FP}^{s}\to U_{FP}^{d}\right\}=\left\{\sigma_{FP}^{s}\times \left(N_{i}^{pred}/N_{i}^{M}\right)\to \sigma_{FP}^{d}\right\}$$

(12) $$\begin{array}{cc} \{U_{\mathrm{spatial}} & :U_{\mathrm{spatial}}^{s}\to U_{\mathrm{spatial}}^{d}\}\\ \; & =\{dx_{i}+dy_{i}+d\theta_{i}+{\Delta d}_{i}\;\to \left(\zeta_{L}\times U_{L}+\zeta_{D}\times U_{D}+\zeta_{r}\times U_{r}\right)\} \end{array}$$

Subsequently, the Chi-square test was employed in this paper to analyze the correlation between the uncertainty of static elements in perception and dynamic perception factors in distinct scenarios and weather conditions. Utilizing a perception uncertainty threshold, the level of uncertainty was determined. We then tallied the occurrences of scenarios where the uncertainty of dynamic elements in perception and static elements in perception was relatively small, both were relatively large, and one was larger while the other was smaller. The corresponding statistical table settings are shown in Table 1.

Utilizing the information listed above, the correlation between them can be computed using the Chi-square distribution, where $K^{2}$ can be calculated in Equation (13).

(13) $$K^{2}=\frac{N_{total}\times \left(N_{TP}\times N_{TN}-N_{FN}\times N_{FP}\right)}{\left(N_{TP}+N_{FN}\right)\times \left(N_{FP}+N_{TN}\right)\times \left(N_{TP}+N_{FP}\right)\times \left(N_{FN}+N_{TN}\right)}$$

where $N_{total}=N_{TP}+N_{FP}+N_{FN}+N_{TN}$. A larger value of $K^{2}$ indicates a stronger correlation between the uncertainty of dynamic elements in perception and static elements in perception.

If there is a correlation between the perception uncertainty of dynamic and static elements, the uncertainty of static elements can be qualitatively utilized to determine the level of uncertainty of dynamic elements as shown in Table 1 for the online evaluation.

#### 3.3.2. Offline Uncertainty Assessment Model Construction

In order to assess the effectiveness of the online evaluation model, it was necessary to obtain prior values for the uncertainty of dynamic elements in perception. Therefore, an offline evaluation of the uncertainty of dynamic elements in perception was conducted in conjunction with dataset annotations.

To ensure consistency in the matching of perception results, this was accomplished by calculating the IOU of the perception results from a bird’s-eye-view (BEV) perspective. This facilitated the alignment of perception results with data annotation values, followed by statistical computations of the classification and spatial uncertainty.

For each detected object *O*, the predicted result is denoted as $O_{P}^{j}$, and the *i*th result of the dataset’s annotation values is represented as $O_{GT}^{i}$. Every element in both the predicted and annotation values is derived from Equation (14).

(14) $$IOU_{ij}=f\left(O_{GT}^{i},O_{P}^{j}\right)$$

The outcomes of object detection were compared with the annotated values in the dataset, resulting in IOU indicators for each detected object. Since a specific bounding box in the prediction results can potentially overlap with multiple bounding boxes in the dataset annotations, resulting in an intersection over union (IOU) greater than zero, non-maximum suppression (NMS) methods were utilized during the matching process. The detailed steps of the matching algorithm are outlined in Algorithm 3.
**Algorithm 3** Object matching algorithm based on IOU from the perspective of BEV.**Input:** The position coordinates (x,y), size (w,l), and orientation (*r*) of the detected objects       and corresponding dataset annotations. **Output:** The IOU between the detected objects and corresponding dataset annotations, id
      of objects matched.
  1:Construct bounding boxes based on position (x,y) and size (w,l) for a object.  2:Rotate the bounding box based on the yaw angle *r* to obtain the actual position  3:**for** i=1 to *m* **do**  4:   **for** j=1 to *n* **do**  5:     Calculate the $IOU_{ij}$ between the *i* th bounding box $O_{GT}^{i}$ in the dataset annotation and the *j* th bounding box $O_{P}^{j}$ in perception results.  6:   **end for**  7:**end for**  8:For the calculated mtimesn IOU values, output values greater than a certain threshold and corresponding object id  9:Determine whether there is a duplicate in id. If there is a duplicate, delete the object pairing with a smaller IOU value of id.  10:**return** The id,IOU of objects matched.


The uncertainty associated with missed and false detections in perception results can be computed according to metrics such as the number of dataset-labeled objects, the number of detected objects, and the count of matched objects in the current frame. The calculation of these various statistics is expressed in Equation (15):

(15) $$N\left(O\right)=\left\{\begin{array}{c} N_{TP}^{O},\;if\;O\in O_{GT}\;and\;O\in O_{P},\\ N_{FN}^{O},\;if\;O\in O_{GT}\;and\;O\notin O_{P}\\ N_{FP}^{O},\;if\;O\in O_{P}\;and\;O\notin O_{GT} \end{array}\right.$$

where $N_{TP}^{O}$ denotes the number of objects successfully matched between the dataset annotation and perception results. $N_{FN}^{O}$ represents the number of objects in the dataset annotation values that did not successfully match the perception result, indicating the number of missed objects. $N_{FP}^{O}$ signifies the number of objects in the perception result that did not successfully match the dataset annotation values, representing the number of objects falsely detected. From these values, the missed detection rate and false detection rate for each frame scenario can be further calculated.

(16) $$\sigma_{FN}^{d}=\frac{N_{FN}^{O}}{N_{FN}^{O}+N_{TP}^{O}}$$

(17) $$\sigma_{FP}^{d}=\frac{N_{FP}^{O}}{N_{FP}^{O}+N_{TP}^{O}}$$

where $\sigma_{FN}^{d}$ and $\sigma_{FP}^{d}$ represent the missed detection rate and false detection rate of the current frame, directly impacting the safety of AD. For each element *E* in Equation (3), the spatial position, size, and orientation uncertainties of corresponding elements in matched objects can be calculated.

(18) $$U_{E}=U_{GT}^{E}-U_{P}^{E},\;E\in E_{d}$$

Following the resolution of errors in dynamic elements within detected objects, the uncertainties related to position, size, and orientation can be subsequently addressed.

(19) $$U_{L}=U_{x}+U_{y}+U_{z}$$

(20) $$U_{D}=U_{w}+U_{l}+U_{h}$$

At this stage, the evaluation model for false detection, missed detection uncertainty, and spatial uncertainty of dynamic elements in perception is established.

### 3.4. Online Assessment of Perception Uncertainty Considering Weather and Scene Factors

#### 3.4.1. Weather and Scene Detection

In real-world operational scenarios, there exist substantial variations in the size and types of detected objects. When occlusion occurs, the detection performance of small objects and lane lines is significantly compromised. Furthermore, adverse weather conditions can adversely affect the camera’s performance, resulting in varying degrees of degradation in perception results. This effect is particularly pronounced after rainy or snowy weather, due to water accumulation and snow cover. While the detection of dynamic objects remains effective, the perception of static elements such as lane lines is impaired. Hence, scene and weather detection serve as crucial foundations for establishing a correlation between dynamic and static elements in perception. Taking into account the influence of weather and scene factors, the correlation between the uncertainty of dynamic and static elements in perception is heightened.

**Figure 10:**
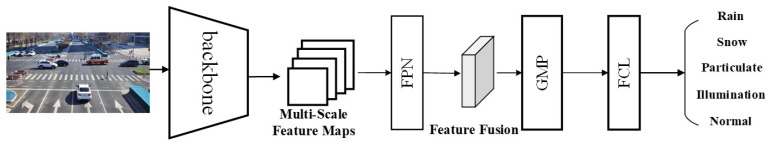
The deep neural network architecture for scene and weather detection.

As shown in Figure 10, the backbone is responsible for extracting and learning features related to scene anomalies such as rain, snow, particulate matter, and lighting variations. The feature pyramid (FPN) addresses the issue of insufficient feature information at different scales by constructing a pyramid-style hierarchical feature structure, enabling the model to better handle objects at various scales. Global max pooling reduces spatial dimensions by taking the maximum value across the entire feature map, allowing the network to focus on the most significant features throughout the feature map. Each neuron in the fully connected layer is connected to all neurons in the preceding layer, forming a fully connected structure used for learning complex features from input data and performing classification. Based on ResNet50, the network ultimately output the classification of five weather scenes.

The dataset was an extension of the SOTIF dataset released by Tsinghua University, including Normal (620 training images + 182 testing images), Rainy (350 training images + 80 testing images), Snowy (335 training images + 82 testing images), Particulate (368 training images + 65 testing images), and Illumination (653 training images + 174 testing images) datasets [37].

According to the test results from the SOTIF dataset, the model achieved an accuracy of approximately 90%. This model can effectively identify weather types and improve the accuracy of the dynamic uncertainty assessment by considering these triggering factors in the correlation of uncertainty between dynamic and static elements in perception. Using the method proposed in this paper, the accuracy of weather and scene recognition is shown in Table 2.

#### 3.4.2. Online Assessment Model Optimization Considering Weather and Scene Factors

When considering weather and scene factors, Equations (9)–(12) in the previous model were further optimized.

(21) $$\alpha =f(U_{d},U_{s},S,W)$$

where S and W denote scene and weather factors, respectively, and α represents the correlation between the uncertainty of dynamic and static elements in perception. S encompasses the number of perception objects, the occurrence of object occlusion, and the presence or absence of lane lines. W includes normal weather conditions, and scenarios involving rain, snow, particulate matter, and illumination.

Specifically, uncertainty relationships of the *i* th frame are established for each perception category as follows:

(22) $$\left\{U_{FN}:U_{FN}^{s}\to U_{FN}^{d}\right\}=\left\{\sigma_{FN}^{s}\times \left(N_{i}^{gt}/N_{i}^{M}\right)+P_{FN}\to \sigma_{FN}^{d}\right\}$$

(23) $$\left\{U_{FP}:U_{FP}^{s}\to U_{FP}^{d}\right\}=\left\{\sigma_{FP}^{s}\times \left(N_{i}^{pred}/N_{i}^{M}\right)\times P_{FP}\to \sigma_{FP}^{d}\right\}$$

where $P_{FN}$ and $P_{FP}$ represent penalty items encountered in adverse operational scenarios.

(24) $$\begin{array}{cc} \{U_{\mathrm{spatial}} & :U_{\mathrm{spatial}}^{s}\to U_{\mathrm{spatial}}^{d}\}\\ \; & =\{dx_{i}+dy_{i}+d\theta_{i}+{\Delta d}_{i}\;\to \left(\zeta_{L}\times U_{L}+\zeta_{D}\times U_{D}+\zeta_{r}\times U_{r}\right)\times P_{\mathrm{spatial}}\} \end{array}$$

where $P_{spatial}$ represents penalty items encountered in adverse operational scenarios.

## 4. Experiment

In order to verify the effectiveness of the uncertainty quantification evaluation in AD perception, we conducted validation experiments based on datasets. The verification goal was to verify the effectiveness of the uncertainty evaluation method of dynamic and static elements in perception by mapping corresponding elements in the HD map, that is, the uncertainty of dynamic elements can be evaluated through the uncertainty of static elements. Based on the above verification goals, the perception uncertainty quantification evaluation dataset validation experiment mainly utilized the nuScenes dataset for the uncertainty evaluation and validation. The verification content was based on the evaluation model validation of perception uncertainty based on the HD map. Specifically, it included evaluating the uncertainty of static and dynamic elements in perception, and evaluating the effectiveness of the method for predicting the uncertainty of dynamic and static elements.

### 4.1. Experiment Settings

(1) Dataset selection

The nuScenes dataset comprises 1000 scenes, encompassing a comprehensive sensor suite for autonomous vehicles, including laser radar and panoramic camera systems, along with detailed annotations of data objects [32]. In computer vision tasks, datasets can achieve object detection and tracking. The entire dataset was annotated with 23 object categories using precise 3D bounding boxes at a frequency of 2 Hz. The dataset can study the generalization of computer vision algorithms in different locations, weather conditions, vehicle types, vegetation, road markings, and left and right hand traffic. The nuScenes dataset contains the map, which contains various static environmental elements that are semantically segmented, enabling the advanced search of relevant data and the retrieval of allocation information labels for any part. In this paper, we selected elements such as road segmentation lines and lane segmentation lines from the dataset map to achieve the semantic segmentation of the map.

(2) Selection of deep neural network algorithms

With the development of perception technology, the perception of AD is moving towards a unified model of multi-task perception. A deep neural network can simultaneously achieve multiple tasks such as semantic segmentation, object detection, and object tracking. This provides a foundation for the correlation of perception dynamic and static element uncertainties based on HD maps. In this paper, we selected the multi-task unified perception model BEVerse deep neural network algorithm to verify the uncertainty evaluation model based on HD map.

The BEVerse algorithm serves as a comprehensive framework for 3D perception and prediction utilizing multi-camera systems. More specifically, BEVerse initiates shared feature extraction and lifting, producing 4D BEV representations from images captured at multiple timestamps and viewpoints [38]. After self-motion compensation, the spatio-temporal encoder was used to further extract BEV features. Finally, multiple task decoders were added for joint inference and prediction. In the decoder, a grid sampler was proposed to generate BEV features that support different ranges and granularity for different tasks. In addition, an iterative flow method was designed to achieve memory-efficient future predictions. In our experiments, we found that time-domain information can improve 3D object detection and semantic graph construction, while multi-tasking learning is implicitly beneficial for motion prediction. Through extensive experiments on the nuScenes dataset, it was shown that the multi-tasking BEVerse framework excels beyond current single-task approaches in multi-perception tasks. Additionally, when compared to sequential processes, BEVerse proves advantageous in substantially enhancing efficiency.

The training implementation platforms were CUDA11.6, CUDNN 8.8, and Python 3.7. We trained the network for five epochs on a Geforce TITAN V GPU with batch size 2. We set the learning rate to 2 × $10^{-4}$.

### 4.2. Dataset Validation Results Based on HD Map

#### 4.2.1. Implementation Details

The dataset validation experiment used the official nuScenes dataset, including the map dataset and camera dataset. The former was mainly used to segment environmental feature elements such as lane lines. The map of the dataset provided the truth values of perception static features, while camera data mainly produced the detection of three-dimensional objects. The visual-based deep neural network algorithm used in the dataset validation experiment belongs to the multi-task unified perception BEVerse model. The internal structure of this network model is consistent in multi-tasking perception, but the output heads for multi-tasking are different. It can achieve functions such as semantic segmentation of lane lines, object detection, and motion prediction. Therefore, it was reasonable to study the correlation between dynamic and static environmental factors based on this model.

During the object detection process, the output of object detection primarily encompasses information such as the semantic category, object position, bounding box dimensions, orientation, and more. The output information comprises spatial three-dimensional details and information derived from the bird’s-eye-view (BEV) perspective. The detected objects encompass a total of 10 categories, namely, cars, trucks, trailers, buses, construction vehicles, bicycles, motorcycles, pedestrians, traffic cones, and traffic barriers. The matching method employed was based on IOU matching from the BEV perspective using deep neural networks. A matching threshold of 0.1 was utilized, and non-maximum suppression (NMS) was applied to ensure the uniqueness of the matching results. The thresholds for determining the correlation between dynamic and static elements in perception are presented in Table 3.

In Table 3, all the thresholds are defined based on statistical results from the nuScenes test dataset; FN and FP represent the missed detection rate and false detection rate for perception dynamic objects, respectively; *S* represents the spatial information of perception objects; *d* and *s* represent dynamic and static elements of perception, respectively; *P* denotes the penalty coefficient, which reflects the punishment on perception performance when there are SOTIF-triggering factors in the weather or scene, aiming to better reflect the objective reality.

#### 4.2.2. Experiment Results

This paper analyzes the experimental results from two perspectives: macro-statistics and specific scene analysis. The analysis included the existence uncertainty and spatial information uncertainty. This paper primarily delves into scrutinizing the correlation between the perception of static factors and dynamic factors through the Chi-square test. It characterizes the relationship between the uncertainty of static and dynamic environmental features. Additionally, the dataset validation experiment scrutinized the correlation between the perception of specific static elements and dynamic elements by integrating perception scene factors and weather factors, offering tailored explanations for the outcomes.

(1) Macro-statistics

Firstly, the validation of the uncertainty in the existence of perception elements was conducted. In the experimental analysis, considering the investigated scenarios, this study selected a total of 63 scenes with 2453 image frames for analyzing the uncertainty of missed detections. Additionally, 61 scenes with 2387 image frames were chosen for analyzing the uncertainty of false detections, and 55 scenes with 2144 image frames were selected for analyzing the spatial uncertainty of the targets. Existential uncertainty is primarily assessed through the statistics of perception-related misses and false detection. The correlation results of perception dynamic and static elements are presented in Table 4 and Table 5.

In terms of quantitative analysis, the Chi-square distribution coefficients computed for misses and false detections through the the Chi-square test were $K^{2}$ = 549.5 and $K^{2}$ = 1428.3, respectively. These values, surpassing 10.828, signify a correlation between static elements in perception and dynamic elements in perception with a 99.9% confidence level.

The uncertainty of spatial information encompasses the spatial information of perception objects. The quantitative results of the the Chi-square test are presented in Table 6.

In terms of quantitative analysis, the Chi-square distribution coefficients computed for the spatial results of detected objects through the Chi-square test was $K^{2}$ = 383.2. These values, surpassing 10.828, signify a correlation between static elements in perception and dynamic elements in perception with a 99.9% confidence level.

Hence, the uncertainty in perception outcomes can be qualitatively assessed and statistically inferred using the static prior information from the HD map. In cases where high uncertainty is identified, preemptive measures should be implemented to ensure the safety of AD.

(2) Specific Scene Analysis

In a particular scenario, the perception of dynamic and static elements was correlated with scene and weather factors, particularly the count of objects in the scene, object occlusion, the absence of lane lines, and weather conditions like night, rain, snow, and fog. Hence, in specific scenarios, a more in-depth analysis can be conducted to explore the correlation between the uncertainty in static element perception and dynamic element perception.

(1) High correlation with good perception effectiveness

On structured roads, the detection of lane lines was ensured if they were complete and well shaped. Additionally, AD scenarios were under normal weather conditions without factors affecting sensor performance When there was no apparent obstruction between the perception of dynamic objects, the detection of dynamic objects was more likely to be ideal. In such scenarios, the uncertainty regarding the existence and spatial information attributes of both static and dynamic elements was relatively low. Therefore, it can be considered that analyzing the uncertainty through the matching of static elements in perception with HD map features reflects the uncertainty of dynamic elements in perception.

**Figure 11:**
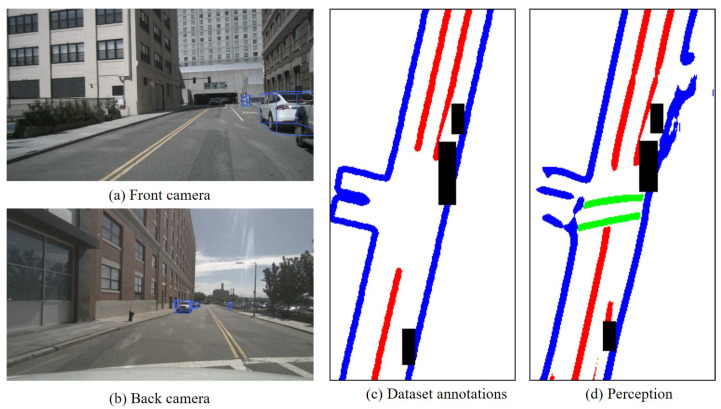
Typical scenarios with good perception effectiveness (Frame 1566).This paper only considers the red lane lines, which represent the static elements. The black boxes represent various dynamic objects.

In the current scenario, we selected a continuous sequence of 40 frames (1565–1604) of images. After conducting the Chi-square test for missed detections, false detection, and spatial uncertainty, the Chi-square distributions were found to be 39.00, 39.00, and 15.90, respectively. This indicates that there is a correlation in the uncertainty of dynamic and static elements in perception in this scene. Figure 11 illustrates a typical scenario of dynamic and static correlation. This scene is the 1566th frame in the nuScenes test set, characterized by a few vehicles, clear lane lines, and no apparent occlusion under normal weather conditions. In fact, as long as there were no significant obstructions or adverse weather conditions, such as SOTIF triggering factors, the perception effectiveness of dynamic and static elements tended to be ideal with a high correlation.

(2) High correlation with poor perception effectiveness

If a large number of objects were detected in a crowded scenario, leading to significant occlusion, the detection of static elements like lane lines was intermittent. In that case, the detection effectiveness between static and dynamic elements was poor. In such a scenario, where there was a high level of uncertainty regarding the existence and spatial information attributes of both static and dynamic elements in perception, it can be considered that analyzing uncertainty through the matching of static elements in perception with high-definition map features reflects the uncertainty of dynamic elements. Typical scenarios with poor perception effectiveness are shown in Figure 11.

In the current scenario, we selected a continuous sequence of 40 frames of images (3131–3170). After conducting the Chi-square test for missed detections, false detection, and spatial uncertainty, the Chi-square distributions were found to be 8.41, 11.24, and 39.00, respectively. This indicates that there is a correlation in the uncertainty of dynamic and static elements in perception in this scene. As shown in Figure 12, there was a large number of objects in the surrounding environment of the ego vehicle, with large object sizes and close distances, leading to severe occlusion. The effectiveness of lane line detection and vehicle object detection by the ego vehicle was poor. The lane lines were sparse, and the figure indicates instances of missed detections in the annotations.

Moreover, if weather conditions such as rain, snow, abnormal lighting, or haze occur in the scene, it could lead to a decline in perception effectiveness for both dynamic and static elements. The uncertainty of dynamic elements in perception could also be reflected through the evaluation of uncertainty in perception static elements.

(3) Low correlation but can be enhanced through post-processing

If weather conditions such as rain, snow, particulate matter, or illumination occur in the scene, there could be a low correlation between the assessment of uncertainty in static elements in perception and the assessment of uncertainty in perception dynamic elements. Therefore, it was necessary to conduct scene detection first. If abnormal weather was identified in the recognized scene, a certain penalty was applied to the perception results of dynamic and static elements, which implies potential shortcomings such as missed detection Based on this adjustment, a reassessment of the correlation in the uncertainty of dynamic and static elements was conducted to establish their relationship.

**Figure 13:**
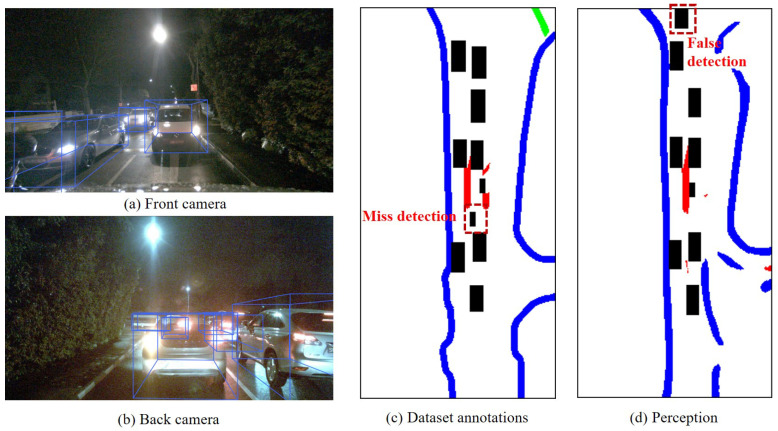
Typical scenarios with poor perception effectiveness (Frame 2206). This paper only considers the red lane lines, which represent the static elements. The black boxes represent various dynamic objects.

In the current scenario, this paper selected a continuous sequence of 40 frames of images (2206–2245). After conducting the Chi-square test for missed detections, false detection, and spatial uncertainty, the Chi-square distributions were found to be 16.25, 11.34, and 12.46, respectively. This indicates that there is a correlation in the uncertainty of dynamic and static elements perception after considering weather and scene. Figure 13 displays a perception scene in low-light conditions. Due to the limited illumination range of the ego vehicle’s headlights, lane lines produced relatively good perception results in the illuminated area. However, in dynamic object detection, the insufficient lightning resulted in poor effectiveness, leading to higher uncertainty. In such situations, penalizing the lane line detection effectiveness could be applied to reflect the perception of dynamic elements. The perception outcomes, after this penalty, became more conservative, as abnormal lighting and adverse weather conditions diminished perception performance, indicating a deficiency in the perception system’s capabilities.

(4) Low correlation but cannot be enhanced through post-processing

If the ground lane lines were lost in the perception scene, the perception effectiveness of static elements was affected. In some long-tail scenarios, such as when the lane line shapes were not sufficiently regular, the detection effectiveness of static elements could decrease. In such cases, it was no longer possible to rely solely on the perception effectiveness of static elements to determine the perception uncertainty. If further assessment of perception uncertainty was needed, additional perception sources were introduced for evaluation. This study was based on the assumption that map information was entirely accurate, and inaccurate scenarios were very rare, which did not impact the overall research on perception uncertainty assessment. Therefore, this aspect is not the focus of this study.

**Figure 14:**
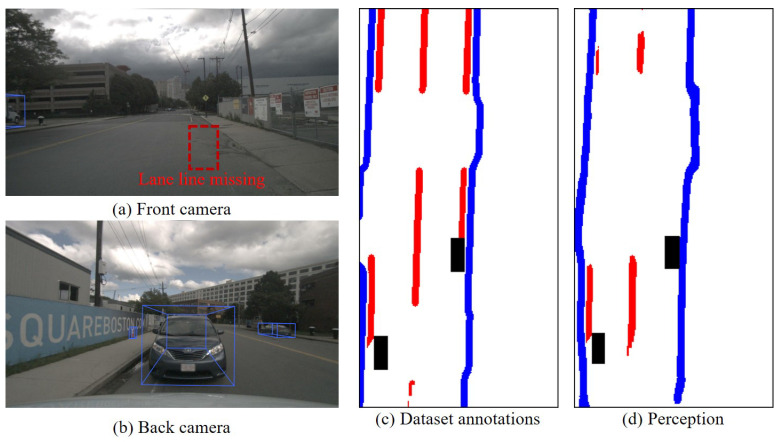
Typical scenarios with poor perception effectiveness (Frame 1355). This paper only considers the red lane lines, which represent the static elements. The black boxes represent various dynamic objects.

Figure 14 illustrates a typical scenario of lane line loss. The HD map displayed complete lane lines, but in actual perception, there was a significant absence of lane lines, leading to high uncertainty after comparing and evaluating lane line detection with the HD map. In fact, this was due to inaccurate updates in prior information, without the influence of factors like weather and occlusion. Additionally, the AD system was unaware of the missing prior information in the HD map. Based on this result, this study concludes that the overall perception effectiveness was poor. Since such scenarios were extremely rare, they did not have a significant impact on the overall perception.

If the perception uncertainty was high, it was necessary to respond to the uncertainty. In this paper, a proactive approach was employed through warning and perception switching, which can meet the real-time safety requirements in AD. On the server used for training the DNN network, the runtime of the uncertainty evaluation algorithm for 1000 frames was 596 s. If parameters are further adjusted and the server’s computational power is increased, the efficiency of the operation can be further improved, thus meeting real-time operational requirements.

## 5. Conclusions

Utilizing prior information from the HD map, this paper establishes an online perception uncertainty assessment method, taking into account environmental and weather factors. In this study, we initially constructed an online uncertainty assessment model for the static elements in perception based on the fusion of topological structure and pixel-level information of lane lines, enhancing the accuracy of uncertainty assessment. Furthermore, an online assessment model for the uncertainty of dynamic elements in perception was also constructed based on the inference of uncertainty from static elements in perception. Then, a deep neural network for weather and scene detection was developed, optimizing and adjusting the online assessment of uncertainty in the perception of dynamic and static elements. This deep neural network achieved an average accuracy of over 90% in recognizing scenes and weather conditions. Utilizing the online uncertainty assessment method, we mapped and evaluated uncertainties such as missed detections, false detections, and spatial uncertainty of perception. Many scenes were selected from the nuScenes test set for the Chi-square test and evaluation, yielding Chi-square ($k^{2}$) values of 549.5, 1428.3, and 383.2, indicating a 99.9% confidence in the existence of correlation in the uncertainty of dynamic and static elements in perception. The proposed online uncertainty assessment method allows for the real-time assessment of perception uncertainty.

Compared to the deep ensemble method, this approach can determine the existence uncertainty of dynamic targets based on the presence indicators of lane lines. In the future, we will incorporate the judgment and classification of occlusion scenes to further enhance the online uncertainty assessment method. Additionally, when lane lines are missing, it is necessary to introduce other perception sources to determine the uncertainty in perception dynamic elements, and even to update prior information of the HD map. In feature selection, additional elements such as lamp posts and signs can be chosen for the uncertainty assessment to compensate for the limitations of relying solely on lane lines perception. The research in this paper will further undergo testing on real vehicles to validate the effectiveness of the algorithm and its real-time operational performance.

## Figures and Tables

**Figure 12:**
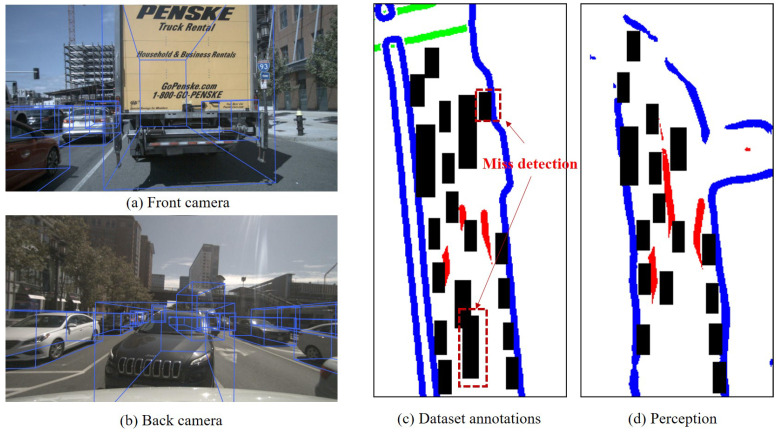
Typical scenarios with poor perception effectiveness (Frame 3132). This paper only considers the red lane lines, which represent the static elements. The black boxes represent various dynamic objects.

**Table 1:** Statistical binomial table for the uncertainty of static elements in perception and dynamic elements in perception.

Uncertainty	$U_{d}$ Low	$U_{d}$ High
$U_{s}$ Low	$N_{TP}$	$N_{FP}$
$U_{s}$ High	$N_{FN}$	$N_{TN}$

**Table 2:** The statistical results of the accuracy in weather scene detection.

Scenes	Normal	Rain	Snow	Particulate	Illumination
Precision	99.97%	85.20%	89.68%	93.1%	94.36%

**Table 3:** The threshold for judging the correlation between dynamic and static perception elements.

Parameters	${\mathrm{FN}}_{s}$	${\mathrm{FN}}_{d}$	${\mathrm{FP}}_{s}$	${\mathrm{FP}}_{d}$	$S_{d}$	$S_{s}$
Threshold	0.46	0.25	0.038	0.25	9	0.8
Parameters	$\zeta_{L}$	$\zeta_{D}$	$\zeta_{r}$	$P_{FN}$	$P_{FP}$	$P_{spatial}$
Threshold	0.7	0.2	0.1	2	0.5	3

**Table 4:** Statistical correlation of missed detections between dynamic and static elements (frames).

	Good Static Perception	Poor Static Perception
Good dynamic Perception	1955	160
Poor dynamic Perception	36	302

**Table 5:** Statistical correlation of false detections between dynamic and static elements (frames).

	Good Static Perception	Poor Static Perception
Good dynamic Perception	2170	138
Poor dynamic Perception	70	9

**Table 6:** Statistical correlation of object spatial information between dynamic and static elements (frames).

	Good Static Perception	Poor Static Perception
Good dynamic Perception	1688	152
Poor dynamic Perception	116	188

## Data Availability

nuScenes dataset: https://www.nuScenes.org/ (accessed on 1 June 2023).

## Abbreviations

The following abbreviations are used in this manuscript:

NMS	Non-Maximum Suppression
CNN	Convolutional Neural Networks
DNN	Deep Neural Network
DE	Deep Ensemble
DBSCAN	Density-Based Spatial Clustering of Applications with Noise
HD map	High-Definition Map
BEV	Bird’s Eye View
SVM	Support Vector Machines
AD	Autonomous Driving
MCD	Monte Carlo Dropout
MCMC	Markov Chain Monte Carlo
IOU	Intersection Over Union
FS	Functional Safety
SOTIF	Safety of the Intended Functionality
KNN	K-Nearest Neighbors
RNN	Recurrent Neural Networks
RANSAC	Density-Based Spatial Clustering of Applications with Noise
TP	True Positive
FP	False Positive
FN	False Negative
